# A Novel Subgroup of UCHL1-Related Cancers Is Associated with Genomic Instability and Sensitivity to DNA-Damaging Treatment

**DOI:** 10.3390/cancers15061655

**Published:** 2023-03-08

**Authors:** Sebastian Burkart, Christopher Weusthof, Karam Khorani, Sonja Steen, Fabian Stögbauer, Kristian Unger, Julia Hess, Horst Zitzelsberger, Claus Belka, Ina Kurth, Jochen Hess

**Affiliations:** 1 Section Experimental and Translational Head and Neck Oncology, Department of Otolaryngology, Head and Neck Surgery, University Hospital Heidelberg, 69120 Heidelberg, Germany; 2 Department of Oral and Maxillofacial Surgery, University Hospital Heidelberg, 69120 Heidelberg, Germany; 3 Tissue Bank of the National Center for Tumor Diseases (NCT), Institute of Pathology, University Hospital Heidelberg, 69120 Heidelberg, Germany; 4 Research Unit Radiation Cytogenetics, Helmholtz Zentrum München, German Research Center for Environmental Health GmbH, 85764 Neuherberg, Germany; 5 Clinical Cooperation Group Personalized Radiotherapy in Head and Neck Cancer, Helmholtz Zentrum München, German Research Center for Environmental Health GmbH, 85764 Neuherberg, Germany; 6 Department of Radiation Oncology, University Hospital, LMU Munich, 81377 Munich, Germany; 7 German Cancer Consortium (DKTK), Partner Site Munich, 81377 Munich, Germany; 8 Division of Radiooncology-Radiobiology, German Cancer Research Center (DKFZ), 69120 Heidelberg, Germany; 9 Research Group Molecular Mechanisms of Head and Neck Tumors, German Cancer Research Center (DKFZ), 69120 Heidelberg, Germany

**Keywords:** ubiquitin C-terminal hydrolase L1, radiotherapy, head and neck squamous cell carcinoma (HNSC, HNSCC), pan-cancer, integrative multi-omics analysis

## Abstract

**Simple Summary:**

In this study, a new subgroup of UCHL1-related cancers was identified across multiple solid tumor entities and was characterized by comprehensive multi-omics analysis. Common features of UCHL1-related cancers are an increased genomic instability and a vulnerability to DNA-damaging treatment, which is accompanied by a favorable prognosis after radiotherapy. A novel classification model has been established for accurate identification of UCHL1-related cancers, leveraging further basic and translational research on this novel cancer subgroup.

**Abstract:**

Purpose: Identification of molecularly-defined cancer subgroups and targeting tumor-specific vulnerabilities have a strong potential to improve treatment response and patient outcomes but remain an unmet challenge of high clinical relevance, especially in head and neck squamous cell carcinoma (HNSC). Experimental design: We established a UCHL1-related gene set to identify and molecularly characterize a UCHL1-related subgroup within TCGA-HNSC by integrative analysis of multi-omics data. An extreme gradient boosting model was trained on TCGA-HNSC based on GSVA scores for gene sets of the MSigDB to robustly predict UCHL1-related cancers in other solid tumors and cancer cell lines derived thereof. Potential vulnerabilities of UCHL1-related cancer cells were elucidated by an in-silico drug screening approach. Results: We established a 497-gene set, which stratified the TCGA-HNSC cohort into distinct subgroups with a UCHL1-related or other phenotype. UCHL1-related HNSC were characterized by higher frequencies of genomic alterations, which was also evident for UCHL1-related cancers of other solid tumors predicted by the classification model. These data indicated an impaired maintenance of genomic integrity and vulnerability for DNA-damaging treatment, which was supported by a favorable prognosis of UCHL1-related tumors after radiotherapy, and a higher sensitivity of UCHL1-related cancer cells to irradiation or DNA-damaging compounds (e.g., Oxaliplatin). Conclusion: Our study established UCHL1-related cancers as a novel subgroup across most solid tumor entities with a unique molecular phenotype and DNA-damaging treatment as a specific vulnerability, which requires further proof-of-concept in pre-clinical models and future clinical trials.

## 1. Introduction

Head and neck squamous cell carcinoma (HNSC) are among the most frequent and destructive human cancers worldwide, causing considerable morbidity and mortality [1,2]. They originate from the mucosal epithelia of complex anatomical structures with smoking, alcohol consumption and high-risk human papilloma virus (HPV) infection as main etiological risk factors. Though a pronounced heterogeneity for HNSC is well established, current treatment protocols consist mainly of surgery and (chemo-) radiotherapy [3]. The therapy is accompanied by significant toxicity and treatment-related morbidity, reducing quality of life. Newly developed targeted therapies considering individual molecular alterations (e.g., EGFR) and the implementation of immune checkpoint inhibition have revolutionized the treatment for patients with recurrent or metastatic HNSC (R/M-HNSC) [4]. However, the objective response rate is low and the number of patients with durable benefit is limited.

Individualization of cancer treatment with the aim of targeting tumor-specific vulnerabilities remains an unmet medical need of high clinical relevance for HNSC and critically depends on accurate stratification of patient subgroups with unique cellular and molecular profiles. HPV-positive oropharyngeal SCC (OPSCC) are the paradigm for this concept, representing a distinct tumor entity within HNSC with different cellular and molecular traits and clinical features. The latter includes a higher sensitivity to radiotherapy and a favorable prognosis [5,6]. Consequently, therapy de-escalation for HPV-positive OPSCC is a vital option to reduce toxicity and treatment-related morbidity by maintaining excellent survival [7]. This highlights the potential of improved subgroup definition and emphasizes that molecular traits underlying cancer cell behavior should be considered for treatment decision.

In this study, we applied comprehensive in silico analysis to describe a new group of UCHL1-related cancers with shared molecular characteristics and vulnerabilities across tumor entities, establishing a foundation for treatment improvements. UCHL1 (ubiquitin C-terminal hydrolase L1) belongs to a subfamily of deubiquitinases (DUBs), which play fundamental roles in numerous biological processes, including cell growth and differentiation, signal transduction, DNA repair and oncogenesis [8,9]. Altered UCHL1 expression and function have been reported in several cancers, providing growing evidence for its contribution to tumor biology [10,11]. However, there is no comprehensive study on the role and function of UCHL1 in HNSC pathogenesis and therapy, so far.

## 2. Materials and Methods

### 2.1. Key Resources

Details on all publicly available data, patient cohorts, online tools, software and algorithms used in this paper are listed in Supplementary Table S1. Patient cohorts used in this study are TCGA-HNSC consisting of 515 primary HNSC, samples from TCGA PanCancer Atlas assembling 9156 other solid tumor types, the Munich cohort with 108 HNSC consisting of 74 primaries and 34 matched primary/recurrent tumors and 1148 established cell lines from CCLE.

### 2.2. Immunohistochemical Staining and Quantification

Paraffin-embedded tissue specimens of primary HNSC from surgical resections of the GSE117973 cohort were provided by the tissue bank of the National Center for Tumor Diseases (NCT, Institute of Pathology, University Hospital Heidelberg, Heidelberg, Germany) and were analyzed according to protocols (ethic votes: S-206/2005 and S-232/2022), approved by the Ethics Committee of Heidelberg University, with written informed consent from all participants. This study was conducted in accordance with the Declaration of Helsinki. Immunohistochemical (IHC) staining with an anti-UCHL1 antibody (HPA005993, Sigma-Aldrich, Taufkirchen, Germany; RRID: AB_1858560) was conducted as described previously [12]. IHC-stained slides were scanned with the VENTANA DP 200 Slide Scanner (Roche, Mannheim, Germany), and semi-automatic quantification of digital images was executed using QuPath version v0.2.3 (RRID:SCR_018257). Artefact regions and invalid samples were manually excluded. For every histological slide, the stain vectors were automatically determined. By using a detection classifier, different cell types were distinguished into tumor, stroma, immune and other cells, and the positive cell detection function was used to quantify the amount of UCHL1-positive cells. The H-Score was computed, reflecting the overall percentage of positive cells and the staining intensity.

### 2.3. UCHL1-Related Gene Set

Samples from TCGA-HNSC (*n* = 515) were divided based on UCHL1 transcript levels. Differentially-expressed genes (DEGs, −1.5 > log2FC > 1.5 and q-value < 0.05) between lower and upper quartiles were calculated using the limma-voom package in RStudio (3.6.1). UCHL1 co-expressed genes (CEGs) were selected based on a Spearman’s ρ (−0.3 > Spearman’s ρ > 0.3 and *p*-value < 0.05). Common genes of the DEGs and CEGs lists were used to generate the UCHL1-related gene set, which was used for unsupervised hierarchical clustering using the wardD2 method with euclidean distance.

### 2.4. Copy Number Analysis

Global genomic alterations were analyzed using the fraction of altered genome. Genomic regions with segment mean values higher 0.2 or lower −0.2 were classified as genomic gain or loss, respectively. Fisher’s exact test was performed using CoNVaQ and visualization was executed with IGV_2.8.0 software (Integrative Genomic Viewer_2.8.0.)

### 2.5. Mutational Landscape

Mutation counts and candidate genes identified by MutSig 2.0 (RRID:SCR_010779) with a q-value < 0.05 and a somatic mutation frequency of at least 5% for TCGA-HNSC were accessed from cBioPortal. Statistically significant differences between subgroups were determined by the chi-square test.

### 2.6. DNA Methylome Analysis

15,000 probes with the highest variance across all samples, excluding probes of sex chromosomes, were used to compute the global beta mean value. High resolution 450K methylation array data for UCHL1 was used to assess epigenetic regulation by methylation of UCHL1 transcription.

### 2.7. Cell Culture

FaDu (RRID:CVCL_1218), Cal27 (RRID:CVCL_1107), SCC4 (RRID:CVCL_1684), SCC9 (RRID:CVCL_1685) and SCC25 (RRID:CVCL_1682) cells were purchased from the American Type Culture Collection (ATCC, https://www.lgcstandards-atcc.org/ (accessed on 21 November 2021) Manassas, VA, USA), and Detroit562 were purchased from Cell Lines Service (CLS, Eppelheim, Germany). All cell lines were maintained in Dulbecco’s Modified Eagle’s Medium (DMEM, Life Technologies, Darmstadt, Germany) and supplemented with 10% fetal bovine serum (Invitrogen, Karlsruhe, Germany), 2  mM L-glutamine and 50  μg/mL penicillin-streptomycin (Sigma-Aldrich, Germany) in humidified and sterile conditions with 6% CO_2_ at 37 °C. Cell cultures were regularly screened to exclude mycoplasma contamination (Venor^®^GeM Classic Mycoplasma Detection Kit, Minerva Biolabs, Berlin, Germany) according to manufacturer’s recommendation. Authentication of all cell lines was confirmed by the Multiplex Human Cell Line Authentication Test (Multiplexion, Heidelberg, Germany, latest update 8 March 2022).

Cancer cells (1 × 10^6^ per dish) were seeded in a 10 cm TC dishes (day 0) and were treated with 0.5–1 µM Decitabine (DAC, Sigma-Aldrich, Germany) or DMSO as control for 2 days (day 1–2). On day 3, cells were harvested and further processed for total RNA extraction.

### 2.8. Quantitative Real-Time RCR Analysis and Western Blot Analysis

Total RNA isolation, quantification and quality control as well as cDNA synthesis were performed as described previously [12]. Quantitative RT-PCR (RQ-PCR) was performed using 10 ng cDNA and the SYBR Green PCR Mix (Thermo Fisher, Karlsruhe, Germany) in the 7900HT Fast Real-Time PCR System (Applied Biosystems, Darmstadt, Germany according to the manufacturers’ instruction. Primers were purchased from Sigma-Aldrich (Germany), and amplification of three reference genes (TBP, LMNB1, ACTB) was used as an internal reference. The cycle of threshold (CT) for UCHL1 was normalized to the geometric mean of CT values for the reference genes using the ΔCT method. For each primer, efficiency was determined by a dilution series (0.005 to 50 ng) of a cDNA mix from all four cell lines. Generation of protein lysates and Western blot analysis was completed as described elsewhere [13]. Supplementary Table S2 summarizes the details on primers and antibodies, which were used for this study.

### 2.9. Classification Model

Hallmark gene sets, curated pathway gene sets (C2) and oncogenic gene sets (C6) from the Molecular Signatures Database (MSigDB) were used and enrichment scores were computed with the GSVA package in RStudio using Gaussion Kernel based on TCGA-HNSC RNA-seq data. Gene set variation analysis (GSVA) is a computational method which allows calculation of enrichment scores (GSVA scores) of user-defined gene sets on a single sample level. To predict UCHL1-related (sub-clusters B1a and B2b) and other tumors (cluster A and sub-cluster B1b), GSVA scores were used for training of a xgbTree model with the caret R package. Cluster B2a was excluded in the training process, due to its strong enrichment for HPV16-positive tumors. A repeated five-fold cross-validation with three repeats was applied for hyperparameter tuning and performance measure. An amount of 80% of the samples were used as a training set and 20% for performance testing at each round using specificity as performance metric. Further details are provided in Supplementary Table S3.

### 2.10. Radiotherapy Response

Clinical data from TCGA were used to select solid tumors with at least 10% of patients treated with radiotherapy (RT) and at least 20% with a predicted UCHL1-related phenotype. A log-rank test was applied for survival analysis. In addition, we applied our classification model to a cohort from Munich with 108 HNSC consisting of 74 primaries and 34 matched primary/recurrent tumors [14]. Overall tumor recurrence within 24 months after RT was analyzed from 108 primary HNSCC. Time to locoregional recurrence was compared for samples with local recurrence (*n* = 38).

Established cancer cell lines from CCLE were stratified by the classification model (Supplementary Table S4), and differences between cells resembling a UCHL1-related or other phenotype were explored based on survival data reported previously [15].

### 2.11. In Silico Drug Sensitivity Screening

Comprehensive drug screening data for cancer cell lines resembling a UCHL1-related or other phenotype (Supplementary Table S4) were downloaded from the Cancer Dependency Map (DepMap) portal. Viability data from the GDSC study (z-scores) and from the PRISM study (Replicate collapsed log-fold change values relative to DMSO) were analyzed to identify compounds with significant differences between UCHL1-related or other cell lines. In both drug screens, lower scores represent higher drug efficacy. A total of 231 drugs present in both screens were selected for further analysis. Cell line stratification was conducted using the classification model. We compared the mean difference of viability between UCHL1-related and other cancer cells using the Wilcoxon test. Information about the compounds and their mode of action was adapted from the drug treatment metadata of the PRISM screen available at the DepMap portal (www.depmap.org [accessed on 25 December 2022]). At first, the most effective drug classes and candidate drugs were identified from GDSC. Subsequently, we validated this result using PRISM. Drugs with matching direction of mean difference scores and a *p*-value < 0.01 in both screens were regarded as validated.

### 2.12. Statistical Analysis

Statistical analysis was performed in R software (3.6.1) with *p*-values < 0.05 regarded as significant unless otherwise stated.

### 2.13. Data Availability

The data generated in this study are available within the article and its Supplementary Materials files. Publicly available data generated by others were used and listed in Supplementary Table S1.

## 3. Results

### 3.1. Identification of a UCHL1-Related Subgroup in HNSC

**Figure 1:**
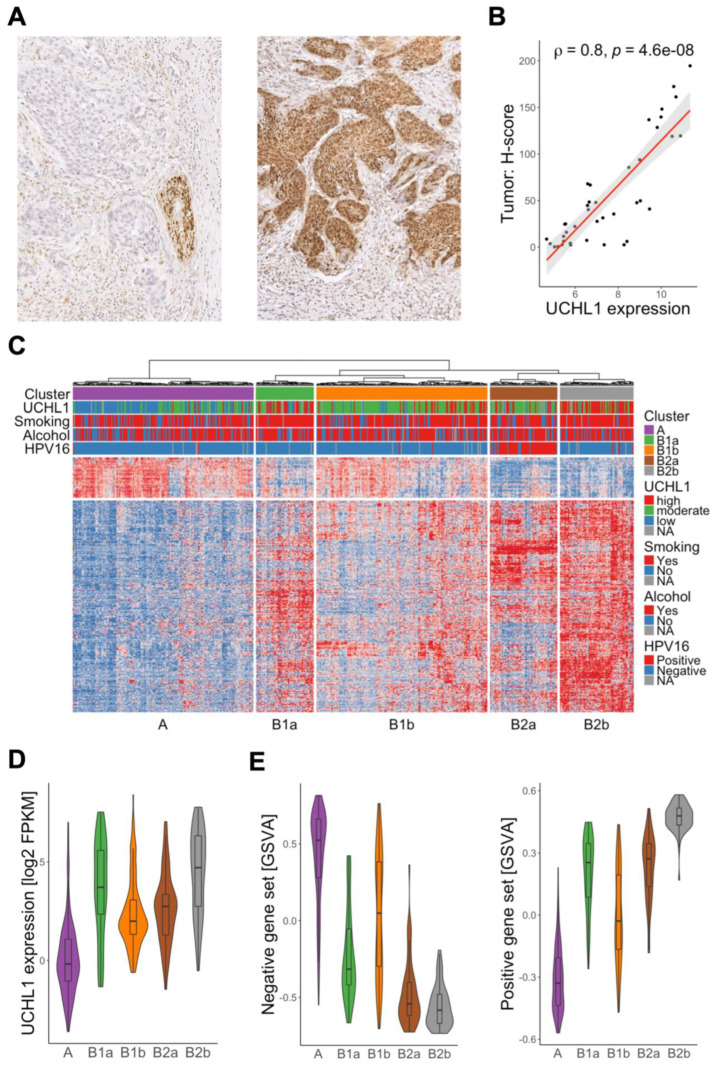
Identification of a UCHL1-related subgroup in HNSC. (**A**) Representative images of an immunohistochemical staining with an anti-UCHL1 antibody on FFPE tumor sections from HNSCs shows a positive staining (brown signal) in peripheral nerves (left) or cancer cells (right). (**B**) Dot plot illustrates the positive correlation between UCHL1 staining intensity (H-score) based on IHC staining and UCHL1 transcript levels for samples of the GSE117973 cohort. (**C**) Heatmap for cases of TCGA-HNSC based on an unsupervised hierarchical clustering with expression data of the UCHL1-related gene set (*n* = 497), applying the wardD2 method and euclidean distance. Violin plots demonstrate variable UCHL1 transcript levels (**D**) and GSVA scores (**E**) for UCHL1-related gene sets with either a negative (left) or positive association (right) to UCHL1 expression in cluster A and sub-clusters B1a, B1b, B2a and B2b.

To detect UCHL1 expression in primary HNSC, we conducted an immunohistochemical staining on tumor sections. In addition to the expected positive staining of peripheral neurons, a prominent staining was also detected for cancer cells in a substantial number of samples (Figure 1A). UCHL1 staining intensity was quantified by QuPath software and was significantly correlated with transcript levels (Figure 1B), confirming a close association between UCHL1 transcript and protein levels and the specificity of the UCHL1 antibody staining. Potential association between UCHL1 expression and histopathological or clinical variables were analyzed by ranking tumors of the TCGA-HNSC cohort according to UCHL1 transcript levels. UCHL1^high^ (top quartile) as compared to UCHL1^low^ tumors (lowest quartile) and UCHL1^moderate^ tumors were significantly associated with a positive smoking history, male sex, alcohol consumption, younger age (<70 years), lymph node metastasis, higher pathological grading and angiolymphatic invasion (Supplementary Table S5).

Differentially-expressed genes (DEGs) and co-expressed genes (CEGs) were identified for TCGA-HNSC to explore molecular differences between tumors with high versus low UCHL1 expression (Supplementary Figure S1A,B). We established a UCHL1-related gene set (*n* = 497) based on common candidate genes of the DEGs and CEGs, consisting of 417 genes with a positive and 80 genes with a negative association to UCHL1 expression (Supplementary Figure S1C). Gene set enrichment analysis confirmed a positive enrichment of gene sets related to UCH proteinases and proteasome degradation and indicated an association with altered immune response and function (Supplementary Figure S1D).

Unsupervised hierarchical clustering of the TCGA-HNSC cohort based on transcript levels of the UCHL1-related 497-gene set revealed two main clusters (A and B) of which the latter was divided into four sub-clusters (B1a-b and B2a-b) (Figure 1C). Sub-clusters B1a, B2a and B2b shared a similar expression pattern of the UCHL1-related 497-gene set, indicating common molecular and functional traits (Figure 1D,E). In contrast, cluster A exhibited an opposite expression pattern of the UCHL1-related 497-gene set and lowest UCHL1 transcript levels, while sub-cluster B1b was more variable, with an intermediate molecular phenotype (Figure 1D,E). It is worth noting that HPV16-positive OPSCC were strongly enriched in sub-cluster B2a (Figure 1C), which had a similar expression pattern of the UCHL1-related 497-gene set as sub-cluster B2b. This indicated elevated UCHL1-related functions in these tumors, despite moderate UCHL1 transcript levels.

In summary, we identified a substantial amount of HNSC with moderate to high UCHL1 expression and a characteristic expression pattern of the UCHL1-related 497-gene set, suggesting a distinct subgroup of tumors which might have particular UCHL1-related molecular features.

### 3.2. Molecular Characterization of UCHL1-Related HNSC

**Figure 2:**
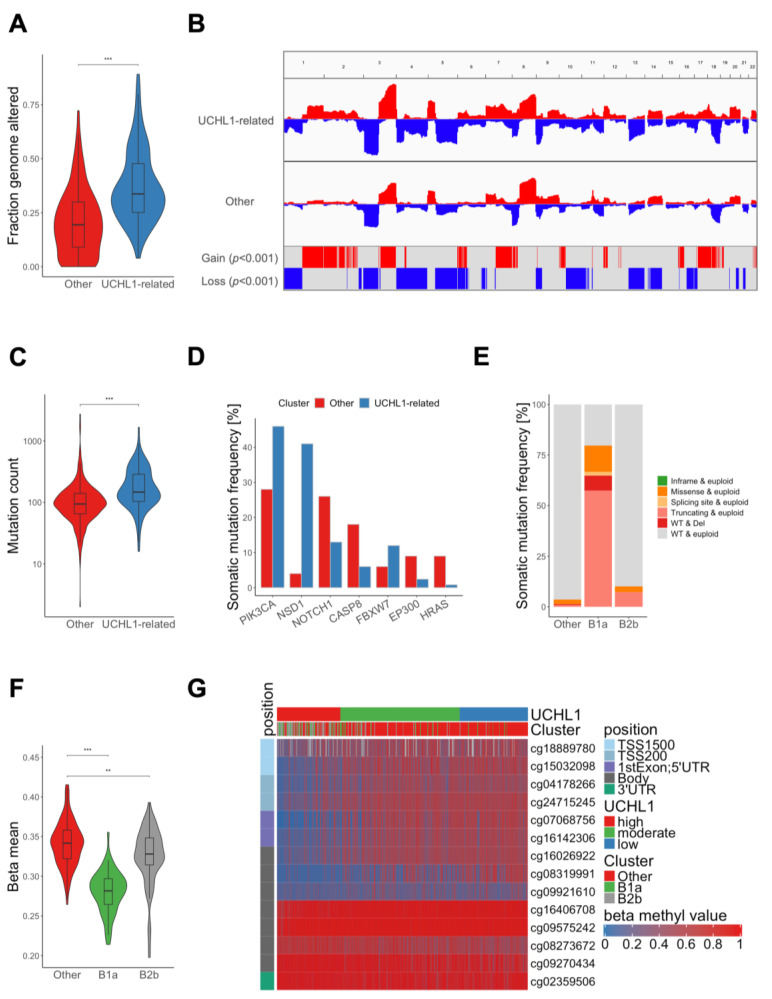
Molecular characterization of UCHL1-related tumors from TCGA-HNSC. (**A**) Violin plot shows a significant difference in the fraction of altered genome between UCHL1-related (blue) and other tumors (red). (**B**) CNV plot illustrates relative frequencies of copy number gains (red) and losses (blue) for UCHL1-related and other tumors with indicated hot spot regions according to a significance level (*p* < 0.001) by a Fisher exact test. (**C**) Violin plot shows a significant difference in somatic mutation counts between UCHL1-related (blue) and other tumors (red). (**D**) Bar plot for MutSig candidate genes with a significant difference (*p* < 0.05) in somatic mutation frequency between UCHL1-related (blue) and other tumors (red). (**E**) Bar plot represents quantitative and qualitative differences in somatic NSD1 mutations for HNSC of sub-cluster B1a, B2b and other tumors. (**F**) Violin plot shows significant differences in global DNA methylation (beta mean) between HNSCs of sub-cluster B1a, B2b and other tumors. (**G**) Heatmap based on supervised clustering (UCHL1 transcript value) with DNA methylation values of probes annotated for the UCHL1 gene locus. ** *p* < 0.005, *** *p* < 0.0005 based on Wilcoxon rank sum test.

We conducted an integrative analysis of multi-omics data from TCGA-HNSC to systematically elucidate molecular differences between UCHL1-related HNSC (sub-cluster B1a and B2b) as compared to other HNSC with either the lowest UCHL1 expression (cluster A) or a heterogenous phenotype (cluster B1b). We excluded tumors of sub-cluster B2a as HPV16-positive OPSCC are considered as distinct tumor entities within HNSC, due to well-established differences in cellular, molecular and clinical features [5,6]. UCHL1-related HNSC showed a significantly higher fraction of genomic alterations (*p*-value < 0.001) with distinct hotspot regions (*p*-value < 0.001) of copy number gains (chromosomes 1q, 2, 3q, 7q, 17, 18) or losses (chromosomes 1p, 3p, 4, 5, 10, 13, 14, 16), indicating an overall genomic instability (Figure 2A,B). In addition, UCHL1-related HNSC possessed an elevated tumor mutational burden (*p* < 0.001) with significant differences in the relative frequency of somatic mutations for selected MutSig genes (q-value < 0.05) of the TCGA-HNSC cohort (Figure 2C,D). One prominent example was a strong enrichment of somatic NSD1 mutations for sub-cluster B1a, including a higher relative fraction of truncating mutations (Figure 2E and Supplementary Figure S2A). NSD1 is a histone methyltransferase, and recent studies demonstrated an association between loss of NSD1 function, in particular by truncating mutations, and a genome-wide hypomethylation in different cancers, including HNSC [16,17]. Indeed, HNSC of sub-cluster B1a exhibited a significantly lower global mean beta value as compared to tumors of sub-cluster B2b (*p*-value < 0.01) or other clusters (*p*-value < 0.001) (Figure 2F). To confirm UCHL1 regulation by global DNA methylation in vitro, established HNSC cell lines with variable UCHL1 expression (Supplementary Figure S2B) were treated with the DNMT inhibitor Decitabine (DAC). Cal27 and FaDu cells with low basal UCHL1 expression showed significantly higher UCHL1 expression after treatment, while no difference was detected for Detroit562 or SCC25 cells with higher basal UCHL1 expression (Supplementary Figure S2C). Finally, DNA methylation values of selected probes (*n* = 7) annotated for the proximal regulatory region of the UCHL1 gene inversely correlated with UCHL1 expression in samples of the TCGA-HNSC cohort (Figure 2G and Supplementary Figure S2D,E). It is worth noting that an inverse association was also evident for tumors of sub-cluster B2b, despite a minor frequency of truncating NSD1 mutations, indicating other molecular modes of epigenetic UCHL1 silencing by DNA methylation (Supplementary Figure S2F).

### 3.3. Prediction of UCHL1-Related Cancers by a Machine Learning Model

Based on single sample gene set variation analysis (GSVA) enrichment scores as model features, we trained a xgbTree classification model for accurate and robust identification of UCHL1-related (clusters B1a and B2b) or other (clusters A and B1b) tumors from TCGA-HNSC. The final parameters and predictive pathway scores (*n* = 354) are shown in Supplementary Table S3. The final model showed a sensitivity of 94.23% and a specificity of 86.96%, measured by repeated cross-validation. Again, sub-cluster B2a was excluded for the training process, due to its strong association with the HPV16 status, but was subsequently classified with the established classification model. As expected, classified UCHL1-related tumors had a significantly higher expression of the 417-gene set with a positive association to UCHL1 expression and a significantly lower expression of the 80-gene set with an inverse association as compared to other tumors (Supplementary Figure S3A), confirming the accuracy of the classification model. Interestingly, many tumors from sub-cluster B2a (41 out of 63) and HPV16-positive HNSCC (35 out of 59) were classified as UCHL1-related, further supporting elevated UCHL1 function, despite moderate expression values, in a substantial amount of HPV16-positive OPSCC (Supplementary Figure S3B,C).

### 3.4. Pan-Cancer Analysis for UCHL1-Related Solid Tumors

**Figure 3:**
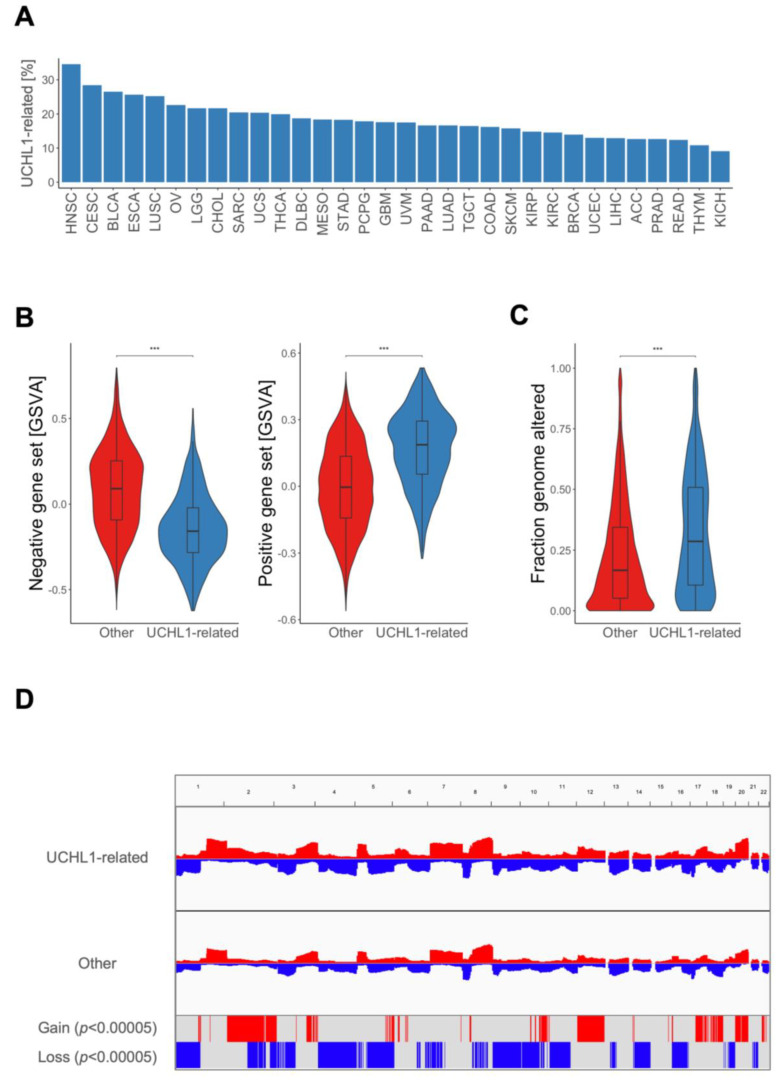
UCHL1-related cancers across solid tumors from TCGA-PanCancer. (**A**) Bar plot indicates relative frequency of UCHL1-related cancers across solid tumor entities of TCGA-PanCancer predicted by the classification model. (**B**) Violin plots show significant differences of GSVA scores for UCHL1-related gene sets with either a negative (left) or positive association (right) to UCHL1 expression between UCHL1-related (blue) and other tumors (red). (**C**) Violin plot shows a significant difference in the fraction of altered genome between UCHL1-related (blue) and other tumors (red). (**D**) CNV plot illustrates relative frequencies of copy number gains (red) and losses (blue) for UCHL1-related and other tumors with indicated hot spot regions according to a significance level (*p* < 0.00005) by a Fisher exact test. *** *p* < 0.0005 based on Wilcoxon rank sum test.

Altered UCHL1 expression and function has been reported across several human cancers [18,19,20,21,22], suggesting the presence of UCHL1-related tumors with similar mutational and molecular landscapes beyond HNSC. This assumption was supported by a positive UCHL1 staining in samples from different solid tumors of the Human Protein Atlas (Supplementary Figure S4). We applied our classification model on samples from the TCGA-PanCancer cohort and identified UCHL1-related tumors across all solid cancers tested (Figure 3A). UCHL1-related cancers from a distinct origin had a similar expression pattern of the UCHL1-related 497-gene set, indicating an accurate prediction for cancers with common molecular features (Figure 3B, Supplementary Figure S5A,B). Moreover, UCHL1-related cancers of the TCGA-PanCancer cohort shared an increased fraction of altered genome (Figure 3C), indicating an overall genomic instability with similar hot spot regions as for TCGA-HNSC (Figure 3D).

### 3.5. Patients with UCHL1-Related Tumors Benefit from Radiotherapy

**Figure 4:**
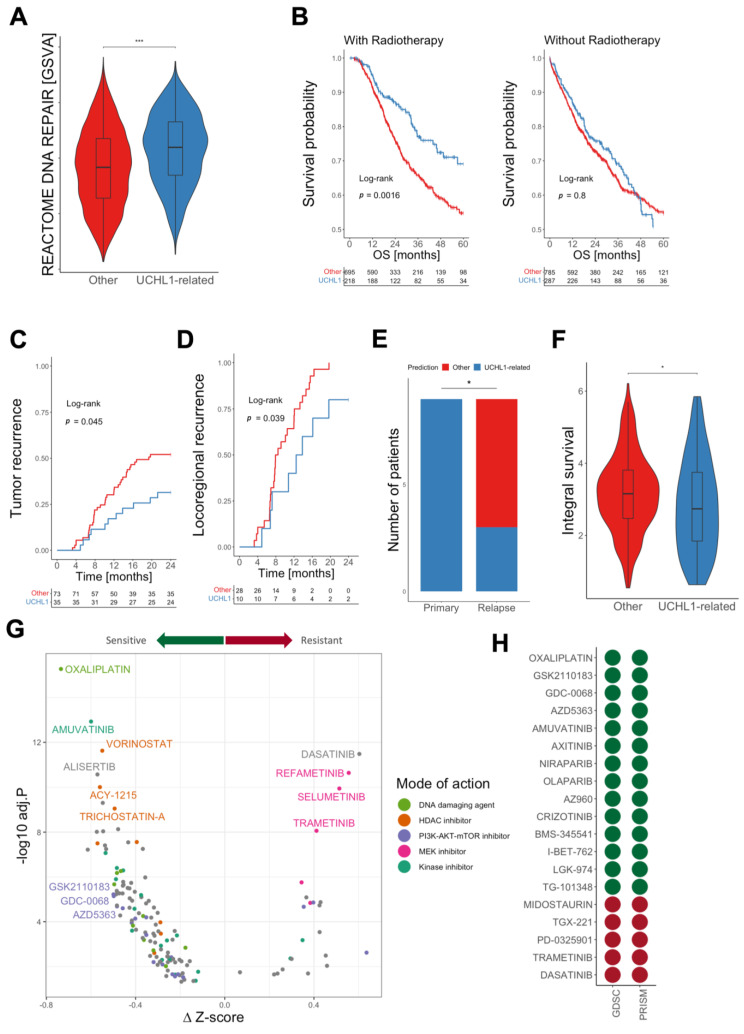
Identification of vulnerabilities for UCHL1-related tumors. (**A**) Violin plot demonstrates significantly higher GSVA scores of the REACTOME DNA REPAIR gene set for UCHL1-related (blue) as compared to other solid tumors (red) from TCGA-PanCancer. (**B**) Kaplan–Meier plots show a favorable five-year overall survival (OS) for UCHL1-related as compared to other solid tumors of TCGA-PanCancer with (left, *p* = 0.0016) but not without (right, *p* = 0.8) radiotherapy. Kaplan–Meier plots show significant differences in overall tumor recurrence (**C**, *p* < 0.045) and time to locoregional recurrence (**D**, *p* < 0.039) within 24 months after treatment between UCHL1-related (blue) and other primary HNSC (red) of the Munich cohort. (**E**) Bar plot illustrates the phenotypic shift from UCHL1-related in primary HNSC to others in matched samples after relapse following radiotherapy in 66% of cases (*n* = 9) of the Munich cohort. (**F**) Violin plot shows a significantly lower integral survival for UCHL1-related (blue) as compared to other cancer cell lines (red) after ionizing irradiation. (**G**) Volcano plot depicts differences in z-scores between UCHL1-related and other cancer cell lines for compounds from the GDSC drug screening project. Minus ∆z-scores indicate a higher sensitivity and positive ∆z-scores a higher resistance of UCHL1-related as compared to other cancer cell lines. (**H**) Summary of compounds with a significant difference (*p* < 0.01) in response rates between UCHL1-related and other cancer cell lines for both the GDSC and PRISM drug screenings, respectively. Color code indicates higher sensitivity (green) or higher resistance (red) of UCHL1-related as compared to other cancer cell lines. * *p* < 0.05, *** *p* < 0.0005 based on Wilcoxon rank sum test. Numbers below Kaplan–Meier plots indicate the patient numbers at risk at the given time points.

An increased genomic instability pointed towards altered DNA damage response and repair pathways, which was supported by a higher GSVA score for the gene set REACTOME DNA REPAIR in UCHL1-related as compared to other tumors of TCGA-PanCancer cohort, including TCGA-HNSC (Figure 4A). Hence, UCHL1-related tumors might be more susceptible to radiotherapy (RT) or other DNA-damaging drugs, which are frequently-applied treatment modalities for most solid cancers. This assumption was further supported by our finding that many HPV16-positive OPSCC with a higher sensitivity to RT shared a UCHL1-related gene expression pattern and were predicted as UCHL1-related tumors by the classification model (Figure 1C, Supplementary Figure S3C). To address this hypothesis, solid cancers from different origins with at least 20% UCHL1-related tumors and at least 10% of cases receiving RT were selected from TCGA-PanCancer (Supplementary Table S6). Within the group of cases with RT, UCHL1-related tumors had a significantly better five-year overall survival (OS) as compared to other tumors, which was not evident for the group of patients without RT (Figure 4B). To further support these findings, we analyzed an independent HNSC cohort (Munich cohort; *n* = 108) with matched pairs of primary tumors and recurrences (*n* = 34) after RT [14]. UCHL1-related primary tumors showed significantly fewer overall events (tumor recurrence) within 24 months (Figure 4C). Within the group of patients with locoregional recurrence (*n* = 38), we observed not only a significant delay in tumor relapse for UCHL1-related HNSC (Figure 4D), but also a shift from UCHL1-related to others phenotype in 66% of cases (Figure 4E). In addition to cancer cell intrinsic traits, variations in the composition of the tumor microenvironment might also explain differences in the RT response. To address this issue, cancer cell lines with publicly available survival data after ionizing irradiation [15] were selected and classified using the classification model (Supplementary Table S4). Again, UCHL1-related cancer cell lines had a significantly higher UCHL1 expression as compared to other cell lines, and we could also confirm significant differences in the UCHL1-related 497-gene set (Supplementary Figure S6A,B). Cancer cells resembling a UCHL1-related phenotype had a significantly lower integral survival after irradiation, confirming an intrinsically higher RT sensitivity (Figure 4F).

### 3.6. Identification of Vulnerabilities by In-Silico Drug Screening

The higher susceptibility to RT indicates specific vulnerabilities of UCHL1-related tumors for genotoxic stress and drugs targeting DNA repair pathways. In line with this assumption, UCHL1-related cancer cell lines had a significantly higher expression of a gene set related to DNA repair pathways (Supplementary Figure S6C). Hence, we conducted an in-silico drug sensitivity screening based on viability data from the GDSC and PRISM projects for selected compound, which were tested in both studies. First, effective compounds with a significant difference in z-scores between UCHL1-related and other cancer cells were identified from GDSC (Supplementary Table S7). This approach revealed a particularly prominent sensitivity of UCHL1-related cancer cells for DNA-damaging agents (e.g., oxaliplatin, carmustine, irinotecan), HDAC inhibitors (e.g., PCI-34051, ACY-1215, vorinostat), PI3K/AKT/mTOR inhibitors (e.g., GSK2110183, GDC-0068, AZD5363) and tyrosine kinase inhibitors, but a higher resistance for MEK inhibitors (Figure 4G). To validate these findings, we analyzed the viability data from the independent PRISM drug screen for UCHL1-related and other cancer cells (Supplementary Table S7). PRISM confirmed a significant difference (*p* < 0.01) in response for 19 compounds, of which the majority (*n* = 14) were more effective in UCHL1-related as compared to other cancer cell lines (Figure 4H). It is worth noting that confirmed compounds included oxaliplatin (DNA damaging), Niraparib and Olaparib (DNA repair pathway). In addition, the list of confirmed compounds was enriched for AKT inhibitors (GSK2110183, GDC-0068, AZD5363), indicating that survival of UCHL1-related cancer cells depends at least in part on PI3K/AKT pathway activity, which appears to be lower as compared to other cancer cells (Supplementary Figure S7A). Subsequently, we confirmed lower PI3K/AKT pathway activity in UCHL1-related tumors from the TCGA-PanCancer cohort (Supplementary Figure S7B).

Based on complementary mechanisms of action, the combination of radiotherapy and certain DNA-damaging drugs or AKT- and PARP-inhibition might be beneficial for patients with UCHL1-related cancers.

## 4. Discussion

Accurate stratification of molecularly-defined cancers with distinct clinical characteristics and vulnerabilities remains a major challenge, but is an attractive vision for more effective and/or less toxic treatment of cancer patients in a personalized manner [7,23,24]. In this study, we demonstrated a prominent UCHL1 expression in cancer cells for a substantial amount of primary HNSC at transcript and protein levels. Aberrantly-expressed UCHL1 has been reported in breast, endometrial, ovarian and lung cancers, emerging as a potential drug target to suppress immune escape, tumor growth and metastasis [18,19,20,21,22,25]. We trained a novel pathway-based machine learning model based on HNSC subgroups with diverse expression patterns of a newly established UCHL1-related 497-gene set to predict a distinct UCHL1-related subgroup within HNSC, but also other solid tumors from TCGA. To date, only limited information on molecular processes causing higher UCHL1 expression or function under physiological or pathophysiological conditions exists from published reports. In this study, we demonstrate global DNA hypomethylation as one mechanism which causes UCHL1 promoter hypomethylation, and consequently, UCHL1 upregulation. In contrast, the rather low frequencies of UCHL1 copy number alterations or somatic mutations across tumor entities formally exclude a strong impact of genetic alterations on UCHL1 expression or function. A common molecular feature of UCHL1-related tumors is a higher frequency of copy number alterations, suggesting a loss of genomic stability. Differences in genomic integrity might be explained by altered stability or activity of key players in genotoxic stress and DNA damage response, which are post-translationally modified by ubiquitin and partially degraded by the ubiquitin proteasome system [26,27].

A highlight of our study is the differences in the survival of cancer patients stratified by the classification model specifically after radiotherapy, indicating an increased radiosensitivity for UCHL1-related tumors. This assumption is supported by the fact that many radiosensitive HPV16-positive HNSC were predicted subsequently as UCHL1-related tumors. It is worth noting that removing from the beginning HPV16-positive tumors of the TCGA-HNSC cohort for establishment of a UCHL1-related gene set has only a minor impact on tumor classification by unsupervised hierarchical clustering and vice versa. The accuracy of the established classification model was high, even when the UCHL1-related gene set based on HPV16-negative tumors from TCGA-HNSC was used for stratification into sub-clusters. A positive association between HPV16 and UCHL1 regulation has been reported previously [28], and investigating the underlying biological principles might elucidate common molecular mechanisms, triggering vulnerability to radiotherapy. Additionally, UCHL1-related HNSC, which had tumor recurrence after radiotherapy, showed a shift from UCHL1-related to the other phenotype, indicating the survival and clonal expansion of RT-resistant cancer cells lacking a UCHL1-related phenotype. These resistant cancer cells either exist prior to treatment or develop due to phenotypic plasticity and might be further elucidated using single-cell approaches. This finding raises the attractive question of whether our established classification model facilitates the stratification of patients with UCHL1-related HNSC for clinical de-escalation trials, including radiotherapy with the aim to improve clinical outcome and quality of life. Our data also suggest that the higher sensitivity to genotoxic stress is, at least in part, cancer-cell-intrinsic as UCHL1-related cell lines from CCLE exhibit a lower integral survival after ionizing irradiation compared to their counterpart without a UCHL1-related phenotype. This is in line with studies reporting resistance to genotoxic chemotherapy in cells from human and C. elegans after UCHL1 downregulation or inhibition of its deubiquitinase activity [29,30]. However, UCHL1 can also stabilize HIF-1, and a UCHL1-HIF-1-mediated metabolic reprogramming has been linked with a radioresistant phenotype [31], emphasizing the need for a better characterization of UCHL1-related cancer cells in adequate pre-clinical models to elucidate underlying molecular principles and potential vulnerabilities. In this context, the in-silico drug screening approach revealed several drugs with a higher efficacy for UCHL1-related cancer cells from CCLE. The list of top ranked candidate compounds included the DNA-damaging agent oxaliplatin and two PARP inhibitors (Niraparib and Olaparib), further supporting the assumption that genotoxic stress represents a vulnerability for UCHL1-related cancers and raising the attractive question of whether UCHL1-related cancers might benefit from combined radio-chemotherapy. In addition, three AKT inhibitors were detected by in silico drug screening. PI3K-AKT pathway activity mediates survival signals and is often related to treatment failure, thus, targeting this pathway is a promising possibility to enhance treatment efficiency [32]. Limited PI3K-AKT signaling might render UCHL1-related cancer cells more sensitive for AKT inhibitors, but also genotoxic drugs or radiotherapy. In line with the in-silico data, up-regulation of AKT phosphorylation has been reported for UCHL1 knockdown cells or in cells with inhibited UCHL1 deubiquitinase activity [30].

A potential limitation of our study is that the classification model depends on global expression data which are currently not available in routine diagnostics. Moreover, the clinical relevance and accuracy of the classification model must be confirmed in a prospective study with a larger cohort of cancer patients with HNSC and also other solid tumors receiving definitive or adjuvant radiotherapy.

In summary, our study elucidated a novel molecularly-defined subgroup of UCHL1-related cancers across solid tumor entities, which is characterized by a higher genomic instability and increased sensitivity to radiotherapy or genotoxic agents as potential treatment vulnerabilities. Insights from this study might support further prospective (pre-) clinical studies investigating UCHL1-related cancers.

## 5. Conclusions

Accurate stratification of patient subgroups with unique cellular and molecular profiles is an unmet medical need of high clinical relevance and enables individualized cancer treatment with the final aim of targeting tumor-specific vulnerabilities. With the identification of UCHL1-related tumors, we established a novel cancer subgroup with distinct molecular traits and favorable prognosis after radiotherapy. Furthermore, the development of a classification model facilitates further investigation of UCHL1-related cancers and their specific vulnerabilities in pre-clinical models and future clinical trials. Thus, this study might leverage basic and translational research on UCHL1-related cancers, contributing to clinical implications improving targeted treatment options for patients with UCHL1-related tumors.

## Data Availability

The data generated in this study are available within the article and its Supplementary Materials files. Publicly available data generated by others were used and listed in Supplementary Table S1.

## Supplementary Materials

The following supporting information can be downloaded at: https://www.mdpi.com/article/10.3390/cancers15061655/s1, Supplementary tables: all tables mentioned in the main text; Source Data Figures: all data underlying the main Figures; Source Data Supplementary Figures: all data underlying the supplementary figures; Supplementary Figure S1: Establishment of the UCHL1-related gene set; Supplementary Figure S2: Molecular characterization of UCHL1-related tumors; Supplementary Figure S3: Evaluation of the ML model for TCGA-HNSC; Supplementary Figure S4: UCHL1 protein expression in selected solid tumors from The Human Protein Atlas; Supplementary Figure S5: Evaluation of the ML model for TCGA PanCancer; Supplementary Figure S6: Evaluation of the ML model for established cancer cell lines from CCLE; Supplementary Figure S7: Enrichment of PI3K-AKT-MTOR signaling in cancer cell lines and tumors from TCGA PanCancer Atlas.
