# Supplementary material for: A Novel Subgroup of UCHL1-Related Cancers Is Associated with Genomic Instability and Sensitivity to DNA-Damaging Treatment
Source: Cancers (Basel). 2023 Mar 8;15(6):1655. doi: 10.3390/cancers15061655 (PMC10099714; doi:10.3390/cancers15061655)
Supplement: Supplementary file 1 [file cancers-15-01655-s001.zip › Supplementary Figure Legends.pdf]

Supplementary Figure S1. Establishment of an UCHL1-related gene set.

Volcano plots for UCHL1 co-expressed genes (CEGs, A) and differentially expressed genes (DEGs, B) between top quartiles of tumors from TCGA-HNSC with highest or lowest UCHL1 transcript levels. Significant CEGs (p-value <0.05) with a positive (Spearman's ρ > 0.3) or negative correlation (-0.3 > Spearman's ρ) concerning UCHL1 expression and significant DEGs (-1.5 > log2FC > 1.5, q-value <0.05) are indicated as blue or red dots. (C) Venn diagrams for CEGs with a positive correlation and up-regulated DEGs (left) or CEGs with a negative correlation and down-regulated DEGs (right) resulting in the UCHL1-related 497-gene set. (D) Graph shows the normalized enrichment scores (NES) based on GSEA with the UCHL1-related 497-gene set for selected gene sets of the MSigDB. Red dots indicate a significant enrichment with the positively associated genes and blue dots a significant enrichment with the negatively associated genes of the UCHL1-related 497-gene set.

Supplementary Figure S2. Molecular characterization of UCHL1-related tumors.

(A) Bar plot represents differences in the somatic mutation frequency for selected candidate MutSig genes between sub-clusters B1a (green), B2b (grey) and other tumors (red) of TCGA-HNSC. (B) Western plot with protein lysate from indicated HNSC cell lines demonstrates variable basal UCHL1 protein expression. Detection of β-Actin served as a control for quantity and quality of protein lysates. (C) Bar plot shows relative UCHL1 expression of indicated HNSC cell lines after treatment with decitabine (DAC) or DMSO as control and quantified by RQ-PCR. Bars represent mean values ±SEM from three independent experiments. (D) Volcano plot shows an inverse association for TCGA-HNSC between UCHL1 transcript and DNA methylation values for probes annotated for the UCHL1 gene locus. Probes (n=7) with a significant inverse association (Spearman ρ < -0.5, p<0.05) are indicated in red. (E) Dot plot demonstrates

an inverse correlation between mean beta values for selected probes (n=7) with UCHL1 expression for TCGA-HNSC. (F) Violin plot shows significant differences in beta mean methylation values of selected probes (n=7) for sub-clusters B1a (green), B2b (grey) and other tumors (red) of TCGA-HNSC. * $p < 0.05$, ** $p < 0.005$, *** $p < 0.0005$ based on Wilcoxon rank sum test.

Supplementary Figure S3. Evaluation of the ML model for TCGA-HNSC.

(A) Violin plots show significant differences of GSVA scores for UCHL1-related gene sets with either a negative (left) or positive association (right) to UCHL1 expression between UCHL1-related (blue) and other tumors (red) from sub-cluster B2a according to prediction by the ML model. Pie charts illustrate the relative frequency of UCHL1-related tumors (blue) in sub-cluster B2a (B) or HPV16-positive HNSC (C).

Supplementary Figure S4. UCHL1 protein expression in selected solid tumors from The Human Protein Atlas (https://www.proteinatlas.org/).

Representative images of an immunohistochemical staining demonstrates UCHL1 protein expression (brown signal) in cancer cells of tumor tissue from cervix (A), bladder (B), ovary (C), and lung cancer (D).

Supplementary Figure S5. Evaluation of the ML model for TCGA PanCancer.

Box plots demonstrate differences of GSVA scores for UCHL1-related gene sets with either a negative (A) or positive association (B) to UCHL1 expression between UCHL1-related (blue) and other tumors (red) of indicated tumor entities according to the prediction by the ML model.

Supplementary Figure S6. Evaluation of the ML model for established cancer cell lines from CCLE.

Violin plots show significant differences in UCHL1 expression (A), GSVA scores for UCHL1-related gene sets with either a negative (left) or positive association (right) to UCHL1 expression (B), GSVA scores for the REACTOME DNA REPAIR gene set (C) between cancer cell lines resembling an UCHL1-related (blue) or other tumor phenotype (red) according to the prediction of the ML model. * p <0.05, ** p<0.005, *** p<0.0005 based on Wilcoxon rank sum test.

Supplementary Figure S7. Enrichment for PI3K-AKT-MTOR signaling in cancer cell lines and tumors from TCGA PanCancer Atlas.

Violin plots show significant differences in the PI3K-AKT-MTOR-SIGNALING between UCHL1-related (blue) and others (red) in cancer cell lines from CCLE (A) and tumors from TCGA PanCancer Atlas (B). * p <0.05, ** p<0.005, *** p<0.0005 based on Wilcoxon rank sum test.
