# Supplementary figures and images for: A Novel Subgroup of UCHL1-Related Cancers Is Associated with Genomic Instability and Sensitivity to DNA-Damaging Treatment
Source: Cancers (Basel). 2023 Mar 8;15(6):1655. doi: 10.3390/cancers15061655 (PMC10099714; doi:10.3390/cancers15061655)

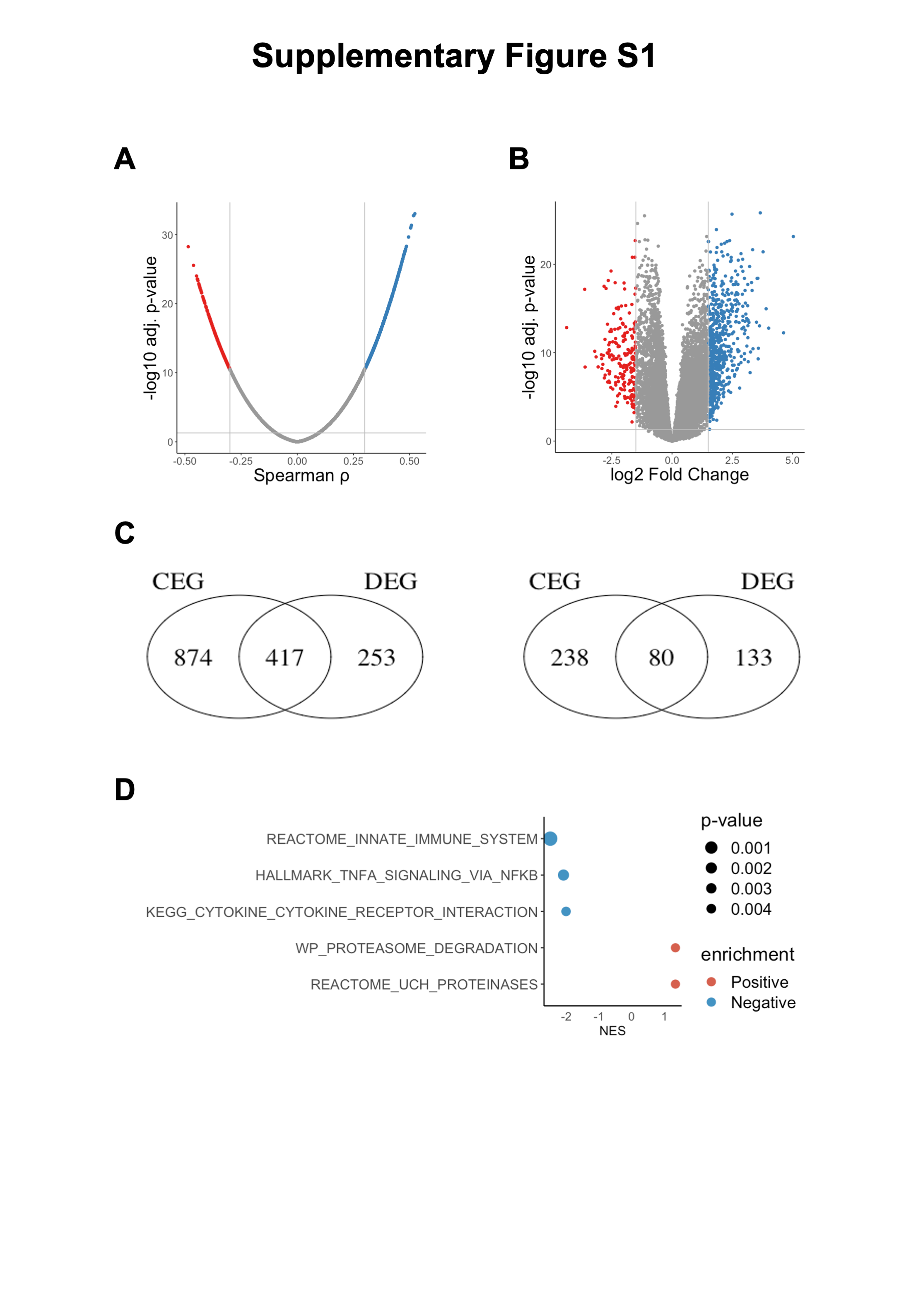

Supplement: Supplementary file 1 [file cancers-15-01655-s001.zip › Supplementary Figure S1.tiff]

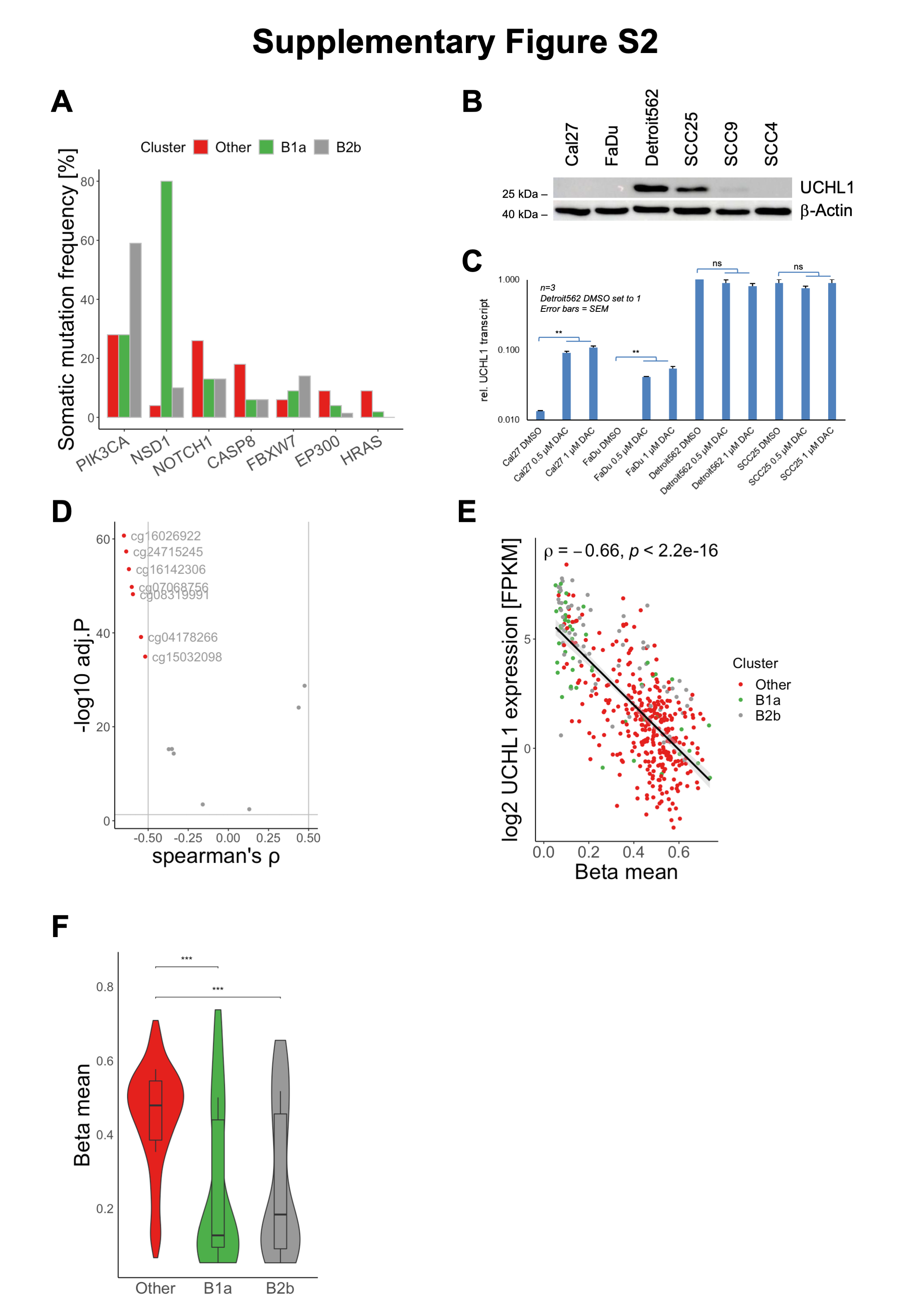

Supplement: Supplementary file 1 [file cancers-15-01655-s001.zip › Supplementary Figure S2.tiff]

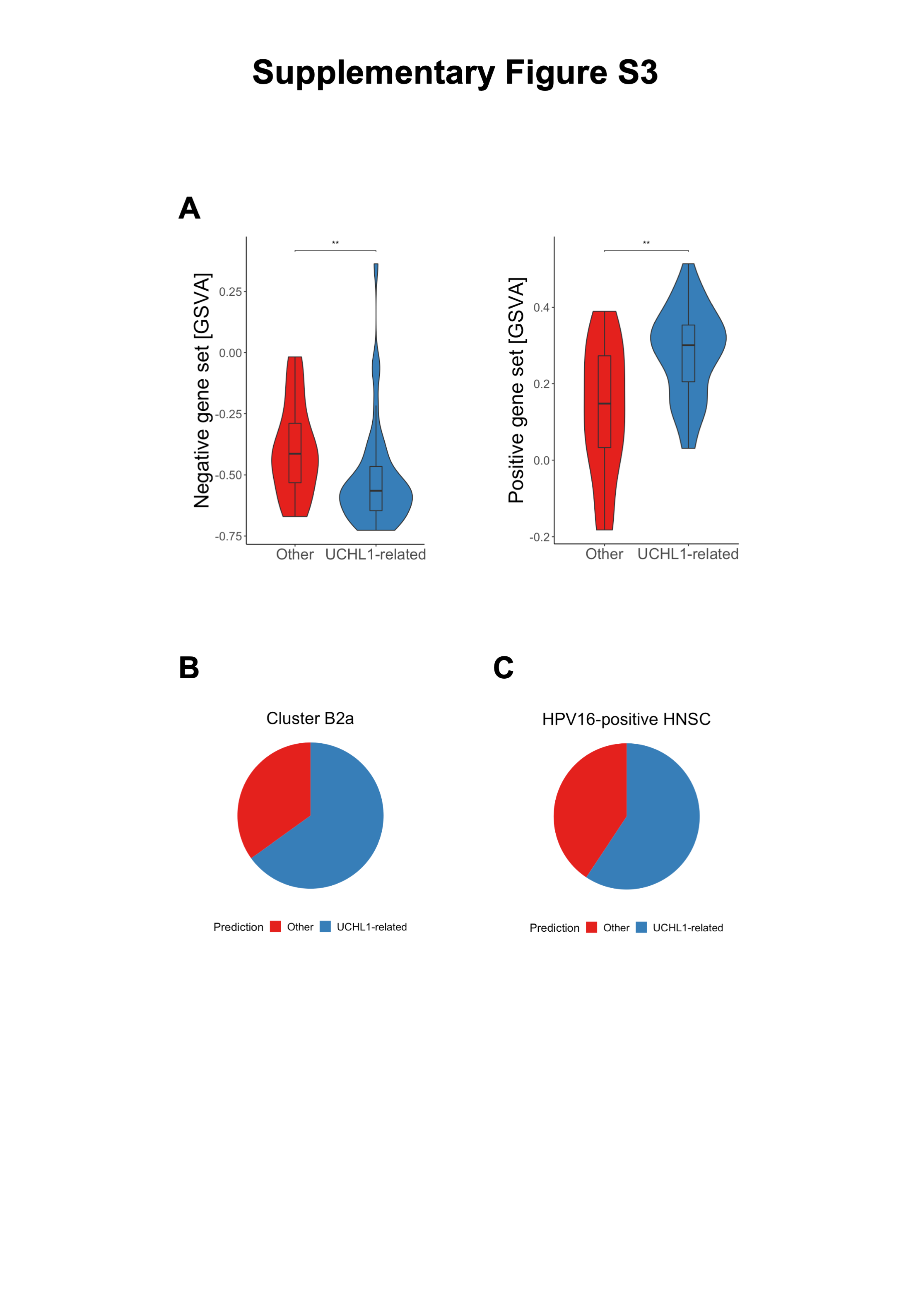

Supplement: Supplementary file 1 [file cancers-15-01655-s001.zip › Supplementary Figure S3.tiff]

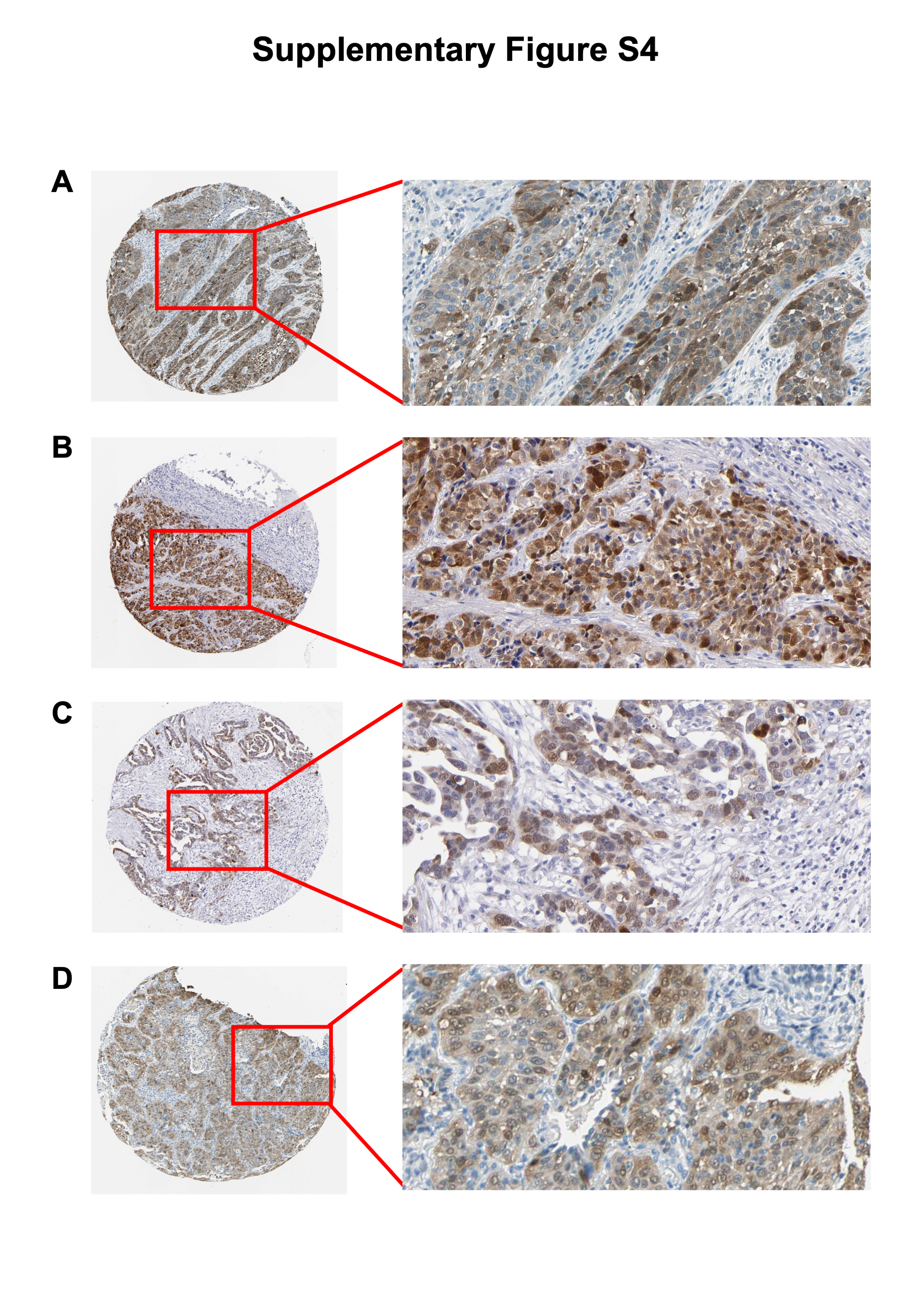

Supplement: Supplementary file 1 [file cancers-15-01655-s001.zip › Supplementary Figure S4.tiff]

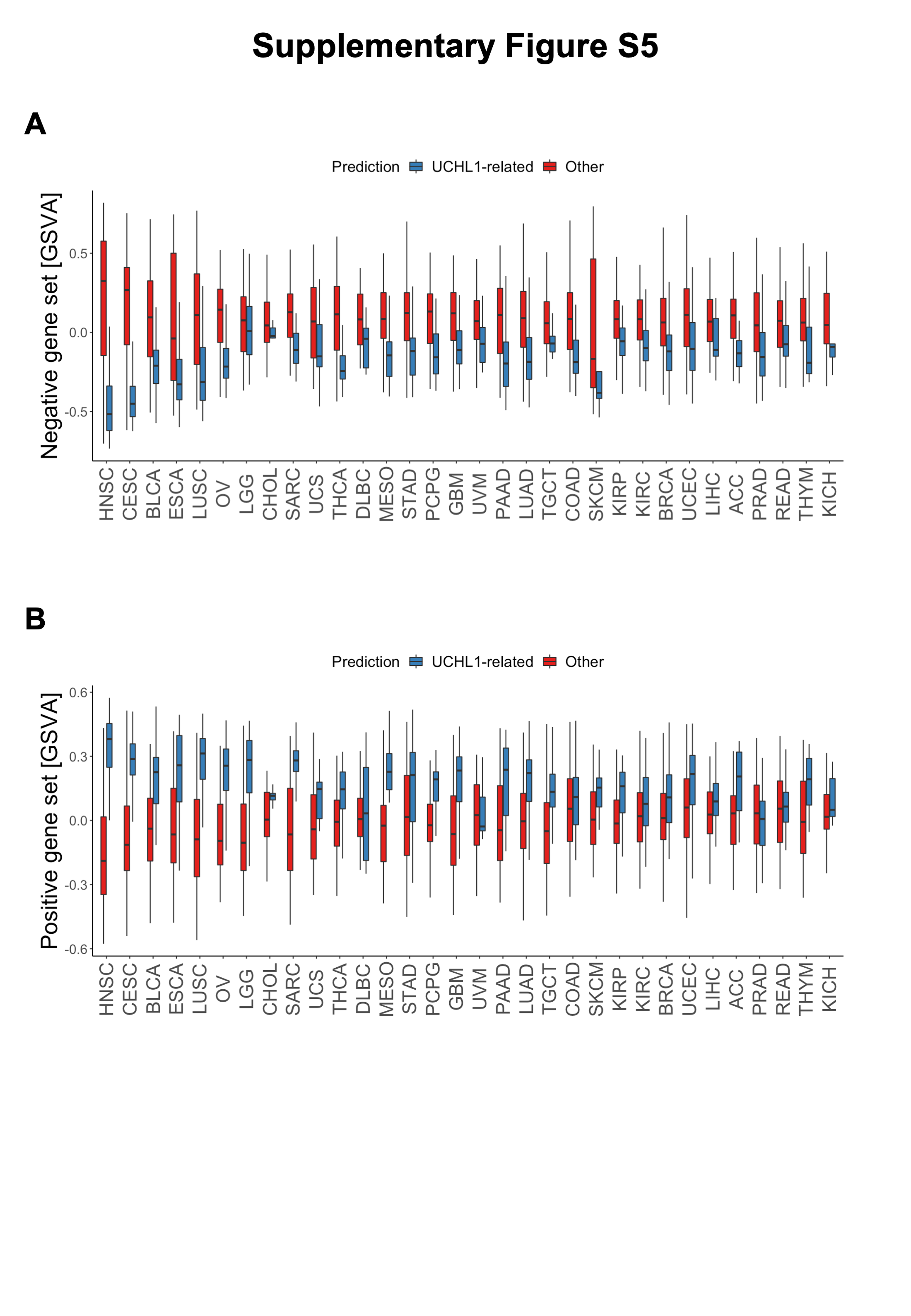

Supplement: Supplementary file 1 [file cancers-15-01655-s001.zip › Supplementary Figure S5.tiff]

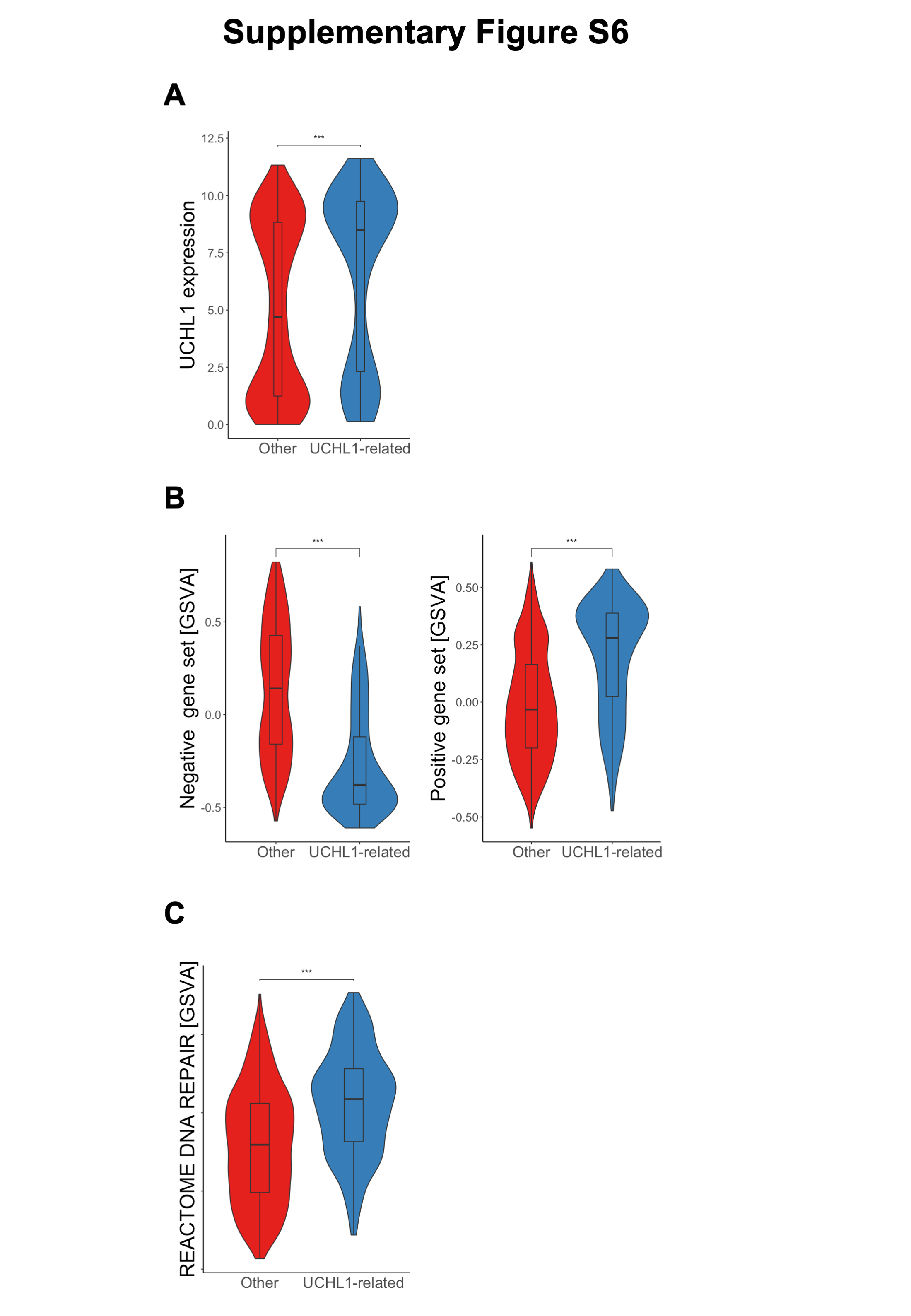

Supplement: Supplementary file 1 [file cancers-15-01655-s001.zip › Supplementary Figure S6.tiff]

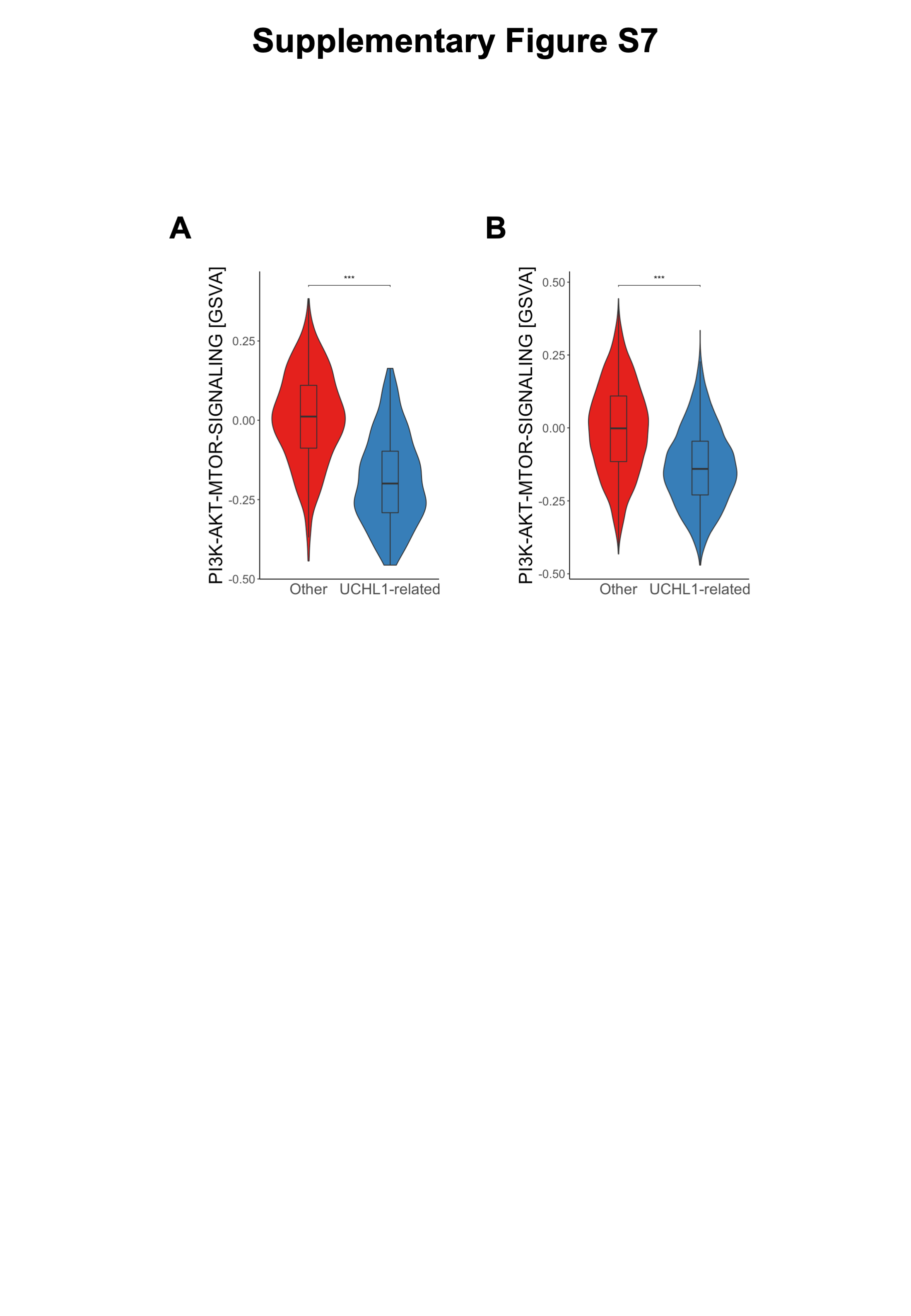

Supplement: Supplementary file 1 [file cancers-15-01655-s001.zip › Supplementary Figure S7.tiff]
